# Unusual Duplication of Flexor Tendons in the Middle Finger Leading to Trigger Finger: A Case Report and Comprehensive Review

**DOI:** 10.7759/cureus.60539

**Published:** 2024-05-18

**Authors:** Christos Lyrtzis, Athina Stamati, Alexandra Brasinika, Konstantinos Stavrothanasopoulos, George Paraskevas

**Affiliations:** 1 Anatomy and Surgical Anatomy, Aristotle University of Thessaloniki, Thessaloniki, GRC; 2 Anatomy and Surgical Anatomy, Medical School, Faculty of Health Sciences, Aristotle University of Thessaloniki, Thessaloniki, GRC

**Keywords:** hand anatomy, trigger finger, anatomical variation, flexor digitorum superficialis, flexor digitorum profundus

## Abstract

Duplication of the flexor digitorum profundus (FDP) tendon is an extremely uncommon anatomical anomaly found within the flexor digitorum superficialis (FDS) muscle, with minimal documentation in the current literature. We present the case of a 45-year-old female manual laborer who exhibited symptoms suggestive of trigger finger in her right middle finger. Surgical exploration uncovered a duplicated FDP tendon, a previously unreported anatomical anomaly in this context. Despite attempting conservative treatment initially, surgical intervention involving release of the A1 pulley, excision of the A1 pulley, and identification of the duplicated tendon was performed. The unusual nature of this anatomical variation highlights the need for additional research into its clinical significance and treatment options. This case highlights the significance of conducting comprehensive anatomical assessments to diagnose and treat uncommon variations within the FDS muscle. It underscores the continued need for collaborative research to enhance treatment approaches, especially in instances where trigger finger symptoms are present.

## Introduction

The intricate variety of movements performed by the human hand is dependent on the exact synchronization of its muscles and tendon [1]. For the fine motor movements that characterize human dexterity, the flexor tendons of the hand are essential [2]. The flexor digitorum profundus (FDP) and flexor digitorum superficialis (FDS), two important muscles, are essential to this complex system [2]. The metacarpophalangeal and distal interphalangeal joints of the index, middle, ring, and little fingers are flexed by the FDP, which is located in the deep volar compartment of the forearm [3]. Its tendons originate from specific points on the forearm and insert into the base of the distal phalanges, allowing for controlled finger flexion [3]. The FDS muscle, located between the superficial and deep muscular layers in the forearm, complements the FDP by allowing for more widespread finger flexion [4]. Innervated by the median nerve and nourished by the ulnar artery, the FDS terminates in a tendinous arch with four tendons reaching the lateral borders of the central phalanx of the second through fifth fingers [5]. Notably, these tendons further divide into the FDS and FDP tendons at their insertion points [4]. The division facilitates intricate finger movements by allowing separate control over the middle and distal phalanges, enhancing dexterity and precision in tasks requiring fine motor skills such as writing or grasping small objects [4].

Understanding their complicated structure is critical for physicians, particularly when dealing with diseases like trigger finger, which impairs the natural gliding of the flexor tendons [6]. While previous literature has documented duplications of the FDS muscle [4,7,8], instances of duplicated FDP tendons are exceptionally scarce [9-11]. In this case report, we aim to provide a detailed anatomical description of a duplicated FDP tendon in the middle finger while also discussing the clinical complexities associated with this uncommon occurrence.

## Case presentation

A 45-year-old female manual worker reported symptoms of soreness at the base of the middle finger and the thumb, snapping with movement, and stiffness consistent with the trigger finger in her right middle finger, prompting a thorough examination of the flexor tendons in the afflicted digit. The symptoms persisted for six months, prompting conservative treatment with nonsteroidal anti-inflammatory medications (NSAIDs) and local corticosteroid injections on both fingers. However, no significant improvements were detected. She also had complaints of pain and burning in the thumb, index finger, and middle finger. The patient also had clinical symptoms of carpal tunnel for the last six months. The nerve conduction velocity test confirmed the diagnosis of carpal tunnel syndrome.

Upon physical examination, the patient showed discomfort at the base of the middle finger and the thumb and a palpable nodule over the A1 pulley. Stiffness and consistent blocking or triggering at the A1 pulley were seen during active and passive finger flexion and extension. The patient described a further increase of symptoms, including discomfort and an inability to completely extend the finger, which required manual aid to straighten. Importantly, there were no concurrent medical conditions such as diabetes, hypokalemia, or rheumatoid arthritis. Due to symptom recurrence and worsening functional limitations, surgical intervention was considered necessary. A 250 mmHg pneumatic arm tourniquet was inserted. Initially, we released the carpal tunnel to relieve pressure on the median nerve, and with another incision, we released the flexor pollicis tendon. Both of them were performed under local anesthesia. Furthermore, a 1.5 mm randomized incision was made just distal to the palmar crease using 2 cc of 2% lidocaine as a local anesthetic. Following longitudinal blunt dissection, the A1 pulley was completely opened with a longitudinal incision, and a cautious resection of roughly 2-3 mm of the A1 pulley was done to reduce the chance of recurrence.

**Figure 1:**
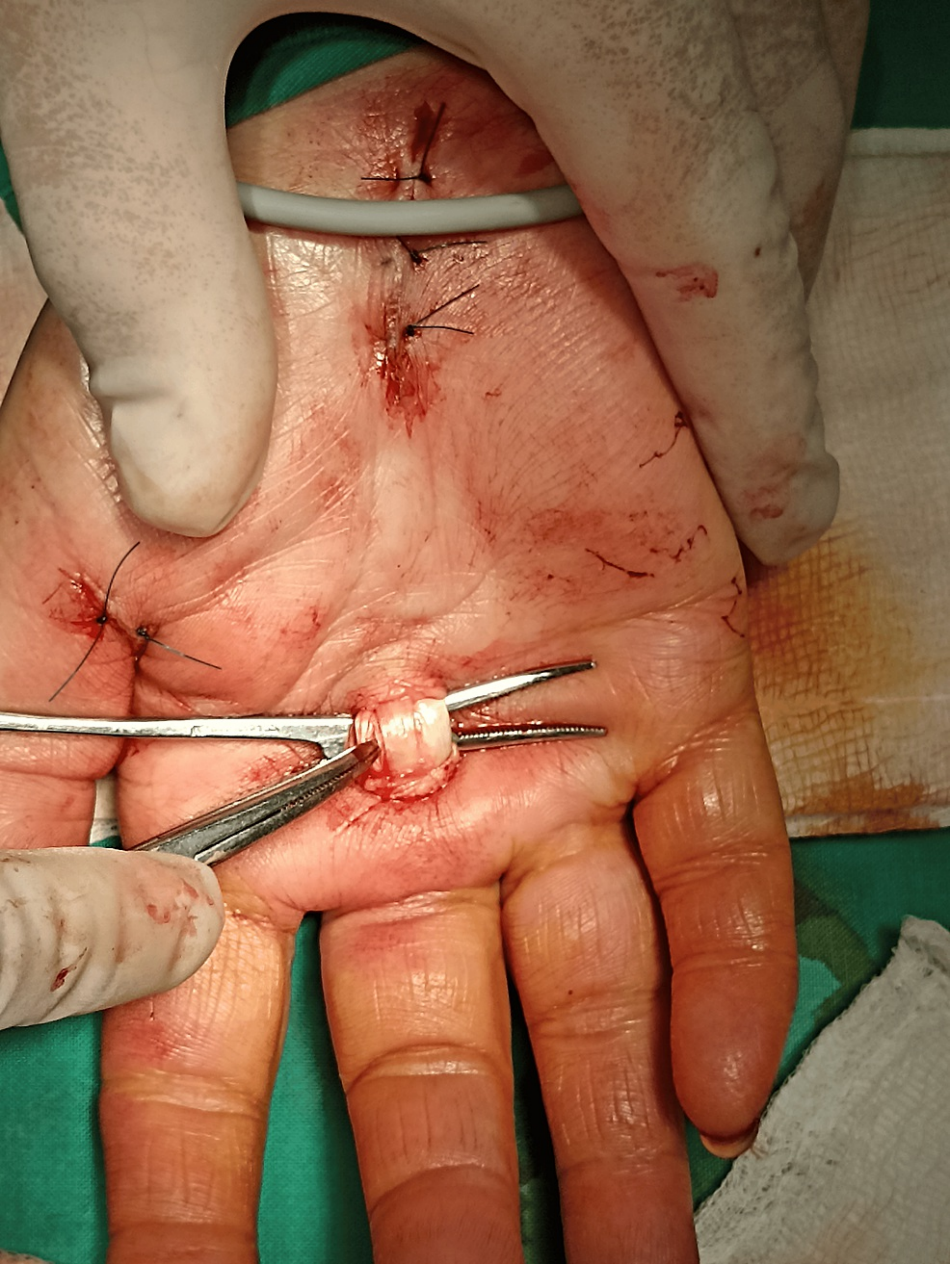
Double FDP tendon of the middle finger. FDP: flexor digitorum profundus.

During the surgical examination, however, a significant discovery was made: three flexor tendons were discovered within the sheath, rather than the expected two (Figure 1). Active movement of the middle finger revealed the existence of two FDP tendons and one FDS tendon. The duplicated FDP tendons were discovered as the etiology of trigger finger syndrome. The surgical technique successfully addressed the A1 pulley constriction, resulting in better symptoms afterward. Following the surgical procedure, the incision site was thoroughly wrapped with Prolene 4-0 to ensure proper wound closure. A circular bandage was put on the hand and lower arm to promote appropriate healing and support the treated region, and the patient was instructed to keep this dressing on for 48 hours after surgery. A coincidental finding was found during the same surgical operation. The nerve conduction velocity test confirmed the diagnosis of carpal tunnel syndrome. This examination of the carpal tunnel showed abnormalities relating to the median nerve. This unexpected discovery led to a comprehensive examination of the median nerve within the carpal tunnel on the same hand, and median nerve release in the carpal tunnel was done because the patient also reported symptoms of median nerve compression. Given the rare anatomical discovery of a duplicated FDP tendon in the middle finger, it is possible that the presence of multiple flexor tendons within the carpal tunnel may lead to median nerve compression [12,13].

## Discussion

The FDP and FDS muscles play a crucial role in hand function by flexing the metacarpophalangeal and distal interphalangeal joints [11]. Hand surgeons often face various anatomic muscle and tendon variants, with special emphasis on the FDS muscle and its tendons due to known variations such as alterations in muscle belly or aberrant musculotendinous connections [12]. The majority of these variations are identified by chance during surgery, are generally asymptomatic, and hence do not require therapy [12]. While magnetic resonance imaging (MRI) can be a diagnostic tool, the absence of symptoms or financial limitations may hinder patients from pursuing this alternative, resulting in the inadvertent identification of such cases [13,14].

Elliot et al.'s categorization method, developed in 1999, distinguishes five categories of variants based on a thorough analysis of more than 30 studies [15]. Type I connects FDS muscle tendons, whereas Type II connects FDS muscle tendons to the flexor retinaculum. Type III shows the existence of a digastric superficial flexor muscle. Type IV indicates a distal extension of the FDS muscle belly. Type V comprises anatomical variants of the FDS muscle in the forearm [15]. Our case, which involved the doubling of the FDP tendon in the middle finger, is classified as a Type V anomaly, indicating an anatomical variation that had not before been reported in the literature. The observed anatomical variance in our instance is consistent with Type V abnormalities, as defined by Elliot et al. in 1999, which involve anatomical differences of the FDS muscle in the forearm [15].

Historical examples published in the literature indicate the rarity of Type V mutations, dating back to 1927 and continuing through later publications, emphasizing the infrequency of such occurrences [9]. Elliot et al. reported the second recorded incidence of Type V anomaly in 1980 [15]. During surgery, an exceptional variation was identified in the FDS muscle situated beneath the median nerve in the forearm and wrist. This atypical muscle exhibited the distinctive characteristic of inducing flexion of the proximal interphalangeal (PIP) joint of the index finger under stress [16]. Moreover, Fromont's 1895 anatomical dissection revealed another notable Type V variation, wherein four anomalous FDS muscles originated from the flexor retinaculum [17]. These muscles extended to the base of the middle phalanx in all four fingers, functioning as flexors of the PIP joint. In the standard anatomy, the FDS muscle in the forearm displayed a distinct configuration, with two tendons connecting to the middle and ring fingers, whereas in the described case, these same tendons were inserted into the flexor retinaculum [17]. Yesilada et al. reported a new Type V abnormality in which no tendons were found after surgery and the well-developed FDS muscle extended to the palm of the hand [18]. Given the range of previously documented Type V variants, the anatomical variance shown in this case is unparalleled in the literature. Its extreme rarity highlights the importance of rigorous observation and extensive inquiry, making it a significant case for continuous scrutiny within the area of hand anatomy.

The embryological cause of documented differences in the FDS muscle, including the given example, remains a focus of continuing investigation [19]. Bhat et al. conducted a systematic review aiming to elucidate the genetic underpinnings of such anatomical anomalies. However, they observed that the precise embryological foundations of these variations remain unidentified [20]. The identified variations in the human FDS muscle have been suggested to involve genetic inheritance, drawing comparisons to muscle and tendon arrangements documented in other species like amphibians. The recurrent nature of these findings, rather than being arbitrary, suggests a potential association with hereditary factors [20]. Notably, the intricacy and numerous anatomical differences in the FDS may be seen as primordial evolutionary traits, reflecting patterns reported in other species [19]. While the genetic features give a reasonable explanation, the exact mechanisms driving the development of anatomical differences in the FDS muscle remain obscure [4]. The lack of an adequate embryological framework to account for these anomalies underlines the need for more study to comprehend the numerous mechanisms that develop the anatomy of the hand. As the study evolves, a fuller knowledge of the embryological foundation for these variances may give useful insights into both normal and aberrant musculoskeletal development, expanding our grasp of hand anatomy and disease.

Furthermore, the statistical rarity of trigger finger incidents involving the middle finger, including just 5% of instances with the index or fifth digit largely involved [13], further highlights the unusual nature of this case. These intricacies, together with the paucity of reported occurrences tying the trigger finger to a doubled tendon, contribute to the remarkable relevance of this finding. As the area of hand surgery improves, this instance promotes more investigation into the complex interplay between anatomical differences and clinical symptoms, opening the door for more sophisticated diagnostic and therapeutic techniques in the arena of hand pathology.

The complexities of the current case raise an important question: Was the trigger finger event caused by the existence of the duplicated tendon, or were these occurrences just coincidental? While current literature describes trigger finger as a result of muscle differences, this case presents a new consideration: the possibility of a doubled FDP tendon. This novel component complicates our knowledge of trigger finger genesis, necessitating a reevaluation of the mechanisms that contribute to this prevalent digital flexor ailment.

## Conclusions

In conclusion, the presented case of a doubled FDP tendon in the middle finger adds a unique dimension to the range of anatomical variants within the FDS muscle. The exceptional rarity of this Type V variant, undocumented in existing literature, underscores its significance and warrants further exploration. The distinct correlation with trigger finger symptoms raises fascinating issues concerning the relationship between structural abnormalities and clinical manifestations. This example makes an important addition to the developing discussion of hand anatomy by underlining the significance of rigorous anatomical investigation in clinical practice. The complexities of trigger finger etiology, particularly in the presence of a duplicated tendon, need a reevaluation of the components that contribute to this prevalent digital flexor ailment. As the field of hand surgery advances, continuing reporting of such instances and joint research efforts are critical for furthering our understanding, refining diagnostic tools, and optimizing therapeutic options for unusual anatomical variants within the flexor tendons.
